# Case of Lodgement of Foreign Body in Air-Passages

**Published:** 1855-12

**Authors:** J. P. Batchelder

**Affiliations:** New York


*History of a Case in which a Foreign Body was Supposed to be Lodged in the Air-Passages—Tracheotomy—Death—Autopsy.* By J. P. BATCHELDER, M. D., etc., of New York.

The following case which is *unique* so far as my knowledge extends, came under my observation several years ago. I took notes of it at the time, but am unable to lay my hand upon them now. I venture, however, to relate from recollection its principal details, for, owing to circumstances connected with it, the case made an impression on my mind, which will probably never be effaced.

A little girl about two years old, while playing in her cradle, was suddenly seized with a most violent cough, accompanied by symptoms of suffocation of the most threatening character. I saw her about half an hour after this occurrence. The symptoms had abated, and the child was comparatively easy. On enquiring into the circumstances which attended the seizure, one of the older children told us that "baby had swallowed a button," with which she had been playing at the time. This seemed to afford a very satisfactory explanation of the phenomena. I explained to the parents the probable nature of the case, the requisite remedy, and the results which would probably ensue with or without its application. A probang was passedd own the œsophagus into the stomach, to remove all doubts as to the direction taken by the extraneous body; and unsuccessful attempts were made to determine, with the aid of the stethoscope, its exact location.

After a short time, the child became tranquil, and soon appeared to be as well as usual. It was therefore deemed proper to postpone any interference until the development of further symptoms.

In a few days the fits of coughing and dyspnœa returned, and recurred with such frequency and severity that it was determined on consultation to advise the operation of tracheotomy. The parents having given their consent, the operation was immediately performed. The trachea was opened without difficulty, but, to our great discomfiture, no button nor any extraneous substance whatever, could be found. Luckily, however, the child was much relieved, and continued perfectly well to all appearances, until the wound in the trachea had entirely closed, and for a few days subsequently. Then, all the distressing symptoms returned. The family would not permit a repetition of the operation. The child soon died.

Twenty hours after death the body was examined. No foreign body could be discovered by the most rigid and minute search. It was noticed however, that the black bronchial glands, as they are commonly called, were all enlarged, and that one in particular, situated immediately at the bifurcation of the trachea, was very large. No other morbid phenomenon could be discovered to which the symptoms might be imputed, and it was presumed accordingly, that these enlarged glands, acting mechanically, had been the cause of all the mischief. It is well known that, in chronic pulmonary affections, these glands, situated in great numbers at the roots of the lungs, often become enlarged and indurated, and productive of much irritation by their pressure on the air tubes. I am not apprized, however, of any other case in which they have caused paroxysms of dyspnœa, so closely simulating the phenomena produced by foreign bodies in the air passages.

I need hardly add that the state of things revealed by the autopsy had not been at all anticipated, although, as has been stated, the most careful auscultation had failed to detect the presence or movements of any extraneous body.

This case, and another, in which an indomitable cough was excited by a tumor distorting or deflecting the trachea,* gave rise to a theory which I have advanced† in relation to the peculiar sensation which precedes coughing.

Regarding the bronchial system as an hollow viscus, with the glottis as its outlet, we find it subject to the same laws of feeling as other organs with passages leading into their interior. In irritation of the bladder, pain is felt at the glans penis; if the stomach is aggrieved, thirst, craving, nausea, and other sensations are experienced, which are referred to the fauces; when the gall-bladder is affected, distress is felt at its termination in the duodenum; so, in whatever part of the bronchial system an irritation is seated, the impression it produces is transferred to the larynx, and the mind perceives a peculiar sensation, as of an extraneous body at the glottis, which excites the act of coughing.

The principle on which all these phenomena depend is the sympathy of feeling and synergy which exist between organs and their outlets.

---

* The latter case is alluded to in a paper published by me in the New York Journal of Medicine, January, 1854, p. 19, note.

† *Loco citato*, p. 19.

Although the presence of a foreign body at the rima glottidis is the simplest physical cause of coughing, the purpose of which is to drive away the offending substance by impelling against it a volume of air, yet this cause is by no means indispensable, since coughing may be produced by whatever affects the passage of air through the bronchi, and more particularly by foreign bodies, or by inflammations, within the trachea or bronchi, lessening the capacity of these tubes.

Reflecting on these facts, in conjunction with the two cases I have cited, I inferred that pressure on the trachea, sufficient to diminish its calibre, would, as well as any other irritation, produce the peculiar sensation which excites coughing. This was found to be true; as any one may satisfy himself by experiments on his own person. Enabled by this expedient to excite the sensation and cough at pleasure, I located the former, after repeated trials, in the posterior part of the larynx, and arrived at the conclusion that it was due to the action of the small muscles situated in the vicinity of the arytenoid cartilages.*—*Va. Med. and Surgical Journal.*

* It is interesting to collate the two remarkable cases adduced by Dr. Batchelder with the observations of Dr. Hugh Ley (*on the Crowing Inspiration of Infants*, London Med. Gaz., Feb. 1834, p. 702) and with Dr. Watson's "conjectural pathology" of whooping cough (*Practice*, 3d Am. ed. p. 55.) The following passage from the Physiological Anatomy of Todd and Bowman has also a bearing on the subject. "The few pathological facts which can be collected of diseased states of the vagi nerves are confirmatory of the conclusions deducible from anatomy and experiment. Several instances are recorded of loss of voice and dyspnœa, symptoms resembling those of chronic laryngitis, caused by the compression of the recurrent by an aneurismal or other tumor. The violent convulsive cough, which accompanies enlarged bronchial glands, is probably due to the irritation of the pulmonary branches of the vagi nerves." C. E.

